# Targeted and untargeted metabolomic approach for GDM diagnosis

**DOI:** 10.3389/fmolb.2022.997436

**Published:** 2023-01-05

**Authors:** Izabela Burzynska-Pedziwiatr, Danuta Dudzik, Anna Sansone, Beata Malachowska, Andrzej Zieleniak, Monika Zurawska-Klis, Carla Ferreri, Chryssostomos Chatgilialoglu, Katarzyna Cypryk, Lucyna A. Wozniak, Michal J. Markuszewski, Malgorzata Bukowiecka-Matusiak

**Affiliations:** ^1^ Laboratory of Metabolomic Studies, Department of Structural Biology, Medical University of Lodz, Lodz, Poland; ^2^ Department of Biopharmaceutics and Pharmacodynamics, Medical University of Gdansk, Gdansk, Poland; ^3^ Consiglio Nazionale delle Ricerche, Institute for the Organic Synthesis and Photoreactivity, Bologna, Italy; ^4^ Department of Biostatistics and Translational Medicine, Medical University of Lodz, Lodz, Poland; ^5^ Department of Nursing and Obstetrics, Medical University of Lodz, Lodz, Poland; ^6^ Department of Clinic Nursing, Medical University of Lodz, Lodz, Poland; ^7^ Department of Diabetology and Metabolic Diseases Lodz, Medical University of Lodz, Lodz, Poland; ^8^ Department of Radiation Oncology, Einstein College of Medicine, Bronx, NY, United States

**Keywords:** GDM, lipidomic profiling, cholesteryl esters, untargeted metabolomics, biomarkers

## Abstract

Gestational diabetes mellitus (GDM) is a disorder which manifests itself for the first time during pregnancy and is mainly connected with glucose metabolism. It is also known that fatty acid profile changes in erythrocyte membranes and plasma could be associated with obesity and insulin resistance. These factors can lead to the development of diabetes. In the reported study, we applied the untargeted analysis of plasma in GDM against standard glucose-tolerant (NGT) women to identify the differences in metabolomic profiles between those groups. We found higher levels of 2-hydroxybutyric and 3-hydroxybutyric acids. Both secondary metabolites are associated with impaired glucose metabolism. However, they are products of different metabolic pathways. Additionally, we applied lipidomic profiling using gas chromatography to examine the fatty acid composition of cholesteryl esters in the plasma of GDM patients. Among the 14 measured fatty acids characterizing the representative plasma lipidomic cluster, myristic, oleic, arachidonic, and α-linoleic acids revealed statistically significant changes. Concentrations of both myristic acid, one of the saturated fatty acids (SFAs), and oleic acid, which belong to monounsaturated fatty acids (MUFAs), tend to decrease in GDM patients. In the case of polyunsaturated fatty acids (PUFAs), some of them tend to increase (e.g., arachidonic), and some of them tend to decrease (e.g., α-linolenic). Based on our results, we postulate the importance of hydroxybutyric acid derivatives, cholesteryl ester composition, and the oleic acid diminution in the pathophysiology of GDM. There are some evidence suggests that the oleic acid can have the protective role in diabetes onset. However, metabolic alterations that lead to the onset of GDM are complex; therefore, further studies are needed to confirm our observations.

## Introduction

Gestational diabetes mellitus (GDM) is a glucose-intolerance disorder diagnosed during pregnancy between 24–27 weeks. It may significantly affect the mother’s health and that of the developing fetus and cause metabolic problems that may occur later in life, e.g., developing type 2 diabetes (T2DM) cardiovascular diseases (CVD), obesity, or hyperlipidemia (Nezami et al., 2010; Plows et al., 2018). Gestational diabetes affects 2%–38% of pregnant women, depending on several factors, with diagnostic criteria and population studies being the most significant ones (Alesi et al., 2021). Moreover, the prevalence of GDM is growing, and 204 million pregnant women were affected with GDM in 2017 (Mdoe et al., 2021). This number is projected to rise to 308 million in 2045, mostly in developing countries (Yahaya et al., 2020).

Currently, the “gold standard” used to diagnose GDM is the fasting plasma glucose (FPG) concentration and an oral glucose-tolerance test (OGTT) which is carried out between the second and the third trimesters (24–28 weeks) of pregnancy. However, some studies suggest that FPG in early pregnancy is not a promising biomarker of GDM (Benhalima et al., 2021; Cosson et al., 2021). On the other hand, OGTT is an inconvenient examination for pregnant women due to its procedure and duration. Drinking the required amount of glucose solution may make some patients feel nauseous (Cosson et al., 2017). Therefore, there is a pressing need to evaluate a new diagnostic or prognostic marker for early developing GDM, which may contribute to improving care for pregnant women at risk of gestational diabetes in the early stages of pregnancy and, subsequently, avoid long-term consequences both for the mother and her child (Brink et al., 2016). The current literature indicates several predictive biomarkers of GDM which may be obtained either as a result of the targeted or untargeted metabolomic analyses of various biofluids.

Our long-term interest as a team has been in the molecular etiology of diabetes mellitus, particularly gestational diabetes mellitus (Wojcik et al., 2014; Wójcik et al., 2015; Wojcik et al., 2016). On the other hand, we have also developed the metabolomics approach, including lipidomics, which can serve as a unique tool that, due to its better perspective, may improve the early prediction of change characteristics of GDM by detecting the early dysregulation in metabolism (Sakurai et al., 2019; Mojsak et al., 2021).

Serum, erythrocytes, and adipose tissue can be tested for fatty acids. It is well understood that FA levels in the serum reflect short-term intake (Kohlmeier, 1995) and are more representative of a subject’s current dietary habits (Bradbury et al., 2011). Recently, we demonstrated lipid profile changes in the erythrocyte membranes of women diagnosed with GDM, reflecting the last 60–90 days of their diet. We demonstrated the changes were mainly related to stearic and cis-vaccenic acids, which were elevated among women with GDM. We postulated that these two fatty acids can act as dual biomarkers of specific saturated fatty acid–monounsaturated fatty acid (SFA–MUFA) conversion processes, catalyzed by Δ-9-desaturase and elongase and that this family of fatty acids may be involved in the pathophysiology of GDM (Bukowiecka-Matusiak et al., 2018). It is interesting to compare the lipid status both in serum and in the erythrocytes and their correlation with GDM onset.

Our previous studies revealed the critical role of arginine in the development of GDM as a potential biomarker used for the early detection of this disease (Burzynska-Pedziwiatr et al., 2020). On the other hand, Dudzik et al. (2017) demonstrated a discriminative power of 2-hydroxybutyrate, 3-hydroxybutyrate, and stearic acid between women diagnosed with GDM and normoglycemic pregnant women. Furthermore, lysoglycerophospholipids are closely associated with pregnant women’s glycemic status. In addition, Dudzik et al. (2014) identified certain metabolites with a strong discriminative potential, namely, lysophosphatidylethanolamines, lysophosphatidylinositols, lysophosphatidic acids, taurine bile acids, and long-chain polyunsaturated fatty acid derivatives.

The metabolism of carbohydrates is closely connected with lipid metabolism pathways. The excess carbohydrates is converted into saturated fatty acids (SFAs) and monounsaturated fatty acids (MUFAs) by the *de novo* lipogenesis process (Ameer et al., 2014), which may play a fundamental role in metabolic regulation and can affect the pathogenesis of diabetes, including GDM (Schwarz et al., 2003). For example, palmitic acid induces inflammation, endoplasmic reticulum stress, and insulin resistance (Schwartz et al., 2010; Sieber et al., 2010). In turn, stearic acid promotes adiposity and, similar to palmitic acid, causes insulin resistance (Anderson et al., 2012; Chu et al., 2013). In contrast, oleic acid prevents palmitate-induced metabolic defects (Salvadó et al., 2013).

Human plasma contains fatty acid derivatives mainly in the form of triacylglycerols (TGs), cholesteryl esters (CEs), and phospholipids (PLs) which are components of lipoproteins. In contrast, adipose tissue primarily comprises triacylglycerols, whereas the red blood cell membrane lipidome is composed mainly of phospholipids (Beynen and Hautvast, 1980; Field et al., 1985; Katan et al., 1991; Katan et al., 1997; Baylin et al., 2002).

Cholesterol exists in two forms in the blood plasma: free cholesterol and cholesteryl esters. Both chemical forms are the constituents of circulating lipoproteins such as low-density lipoprotein (LDL), very low-density lipoprotein (VLDL), or high-density lipoprotein (HDL). Cholesteryl esters are the fractions of plasma lipids involved in lipoprotein metabolism. Most of the cholesterol in the blood exists in an esterified form (Hodson et al., 2008). Conjointly with phospholipids, cholesteryl esters can be helpful as potential lipidomic biomarkers (Ferreri et al., 2016). Sansone et al. (2016) demonstrated an interesting diversity of the cholesteryl esters from the plasma and phospholipids from erythrocyte membrane compositions in a group of morbidly obese people compared to lean subjects. In this study, some fatty acids presented the same trends in both classes of lipids; however, some showed the opposite trend.

The two classes of lipids, namely, phospholipids isolated from the erythrocyte membrane and cholesteryl esters isolated from plasma, are connected through the activity of lecithin–cholesterol acyltransferase (LCAT) which is responsible for the transfer of fatty acids from phospholipids to cholesteryl esters. More importantly, this is one of the emerging pathways involved in human pathologies (Jonas, 2000). Other phospholipids, such as phosphatidylethanolamine, can also participate in the LCAT reaction (Christiaens et al., 2000). Fatty acids in phospholipid membranes and cholesteryl esters from the plasma have a close correlation with the onset of insulin resistance and diabetes (Maulucci et al., 2019).

Plasma cholesterol esters consist of relatively high proportions of n-6 polyunsaturated fatty acids (PUFAs) typically present in phosphatidylcholines, i.e., linoleic acid and arachidonic acid. Therefore, cholesterol esters are strongly associated with membrane turnover *via* HDL and are further transferred to other lipoprotein fractions, LDL and VLDL, in a cholesterol–ester transfer protein (CETP)-catalyzed reaction (Melchiorre et al., 2011; Ferreri and Chatgilialoglu, 2012).

In this work, we report the examination of the fatty acid composition of plasma cholesteryl esters in the second trimester of pregnant women with diagnosed GDM (*n* = 32), juxtaposed with normal glucose-tolerant (NGT) women (*n* = 11) and the comparison of the obtained profile with previous results regarding the analysis of erythrocyte membrane phospholipids in the same group of patients. Additionally, we demonstrated the results of the untargeted metabolomic analysis, the purpose of which was to search for compounds that could constitute potential predictive markers.

## Materials and methods

### Study design

GDM was diagnosed according to the following WHO diagnostic criteria (Ferreri et al., 2016): fasting glucose level at 92 mg/dl (5.1 mmol/L) or 153 mg/dl (8.5 mmol/L) 2 h after 75-g OGTT between 24 and 28 week of gestation, or later if it was not possible during this period. After classification, the control group included 11 healthy pregnant women with normal glucose tolerance (NGT), and the GDM group included 32 women with impaired glycemia (43 patients in total). Blood samples were collected in EDTA-containing tubes at 24–28 weeks of gestation (on the day of the OGTT) for both the NGT and GDM groups as we previously described (Ferreri et al., 2016; Bukowiecka-Matusiak et al., 2018).

The inclusion criteria were described in our earlier paper (Burzynska-Pedziwiatr et al., 2020).

Maternal weight and height in the third trimester of pregnancy were measured using standard equipment, which allowed us to calculate weight gain and pre-pregnancy body mass index (BMI). All blood parameters were as follows: total cholesterol, serum triglyceride (TG), HDL, LDL, glycated hemoglobin (HbA1C), C-reactive protein (CRP), and insulin concentrations were measured according to the standard protocols included in our previous work (Bukowiecka-Matusiak et al., 2018). Indicators of insulin resistance (HOMA-IR) and beta-cell function (HOMA-B) for patients were calculated with the use of the homeostasis model assessment (HOMA) according to the protocol (Matthews et al., 1985).

The study was conducted according to the guidelines of the Declaration of Helsinki and was approved by the Ethical Committee of the Medical University of Lodz (No. K.B./268/15 from 17 February 2015). Informed consent was obtained from all participating subjects.

### Untargeted plasma metabolomics analysis

#### Chemicals

Organic solvents used were as follows: acetonitrile, heptane, and 2-propanol Optima™ LC/MS grade were supplied by Fisher Chemical. Ultrapure water was produced using the Milli-Q Plus 185 system (Millipore, Billerica, MA, United States). O-methoxyamine hydrochloride, sialylation-grade pyridine, BSTFA (N,O-bis(trimethylsilyl)trifluoroacetamide) with 1% TMCS (trimethylchlorosilane), C8–C40 alkane calibration standard mix, C:18 methyl ester, and tricosane analytical standard were purchased from Sigma-Aldrich (St. Louis, MO, United States).

#### Untargeted plasma metabolomics analysis

Metabolomic analyses of plasma from control and GDM women were performed using the GC–MS-based approach. Sample analysis was carried out randomly, and the system’s stability, performance, and reproducibility of the sample treatment procedure were checked with the quality control samples. With the use of quality assurance (QA) criteria, matrix filtration was achieved (Dudzik et al., 2017), enabling selection of a total of 33 metabolites for further data processing.

#### Derivatization protocol

An aliquot of 40 µl plasma was mixed with 120 µl cold acetonitrile (−20°C) and centrifuged (15,400 x*g*, 4°C, 10 min). An aliquot of 100 µl of the supernatant was transferred to a gas chromatography (GC) vial, evaporated to dryness under vacuum (SP Genevac miVac Sample Concentrators—S.P. Scientific, Genevac Ltd., Ipswich, Suffolk, United Kingdom), and derivatized as previously described (Rey-Stolle et al., 2021). In brief, 10 µl O-methoxyamine hydrochloride in pyridine (15 mg/ml) was added to each sample to protect the aldehyde and ketone groups. After 16 h of incubation, 10 µl BSTFA with 1% TMCS was used for silylation (1 h, 70°C). Finally, 100 μl heptane containing 20 ppm tricosane (IS) was added to each vial. Quality control (QC) samples were independently prepared by pooling equal volumes of each sample and following the same extraction procedure applied to the experimental samples. Analyte-free extraction blank and reagent blank were also processed (Dudzik et al., 2018).

### GC-Q-MS analysis

The samples (2 μl) were injected in split mode with a 1:10 flow into an Agilent Technologies 8860 gas chromatography system coupled with an Agilent Technologies 5977B mass spectrometer (Agilent Technologies, Waldbronn, Germany). Separation of the metabolites was performed on an HP-5MS capillary column DB5–MS (30 m length, .25 mm i.d., and .25 μm film 95% dimethyl/5% diphenyl polysiloxanes) with an integrated pre-column (10 m J&W, Agilent). The carrier gas (He) flow rate through the column was set at .674 ml/min. The injector port was maintained at 250°C. The temperature gradient was programmed as follows: The initial oven temperature was set at 60°C (held for 1 min) with a ramping rate of 10°C/min up to 325°C and maintained for 10 min before cooling down with the total run time of 37.5 min per sample. The detector transfer line, the filament source, and the quadrupole temperature were set to 280°C, 250°C, and 150°C, respectively. MS detection was performed in the electron impact (EI) mode at a fragmentation voltage of 70 eV. The mass spectrometer was operated in the scan mode with a mass range of *m/z* 50–600 at a rate of 2.7 scans/s. A C:18 methyl ester, C8–C40 alkane calibration standard mix, blanks, and QCs for system equilibration were injected at the beginning of the analytical batch, following QC injections for every six experimental samples.

#### Data processing and compound identification

Acquired data were inspected to assess the data quality with Agilent MassHunter Qualitative Analysis Ver. B.08.00 (Agilent Technologies, Santa Clara, CA, United States). Spectral de-convolution was performed with the Agilent Unknown Analysis tool (Ver. B.08.00. Agilent Technologies, Santa Clara, CA, United States). Alignment of the retention time drift was performed with Mass Profiler Professional ver. B.12.1 (Agilent Technologies, Santa Clara, CA, United States) software. Assignment of the target ion and the qualifiers, entire batch pre-processing, and manual inspection of the data, including the peak area and RT integration, were performed using Agilent MassHunter Quantitative Analysis (Ver. B.08.00, Agilent Technologies, Santa Clara, CA, United States). Compound identification was performed with the NIST (National Institute of Standards and Technology, Gaithersburg, MD, United States) mass spectra library (Ver. 2017). Raw data have been cleared of unrelated features and contaminants.

#### Data treatment

Quality control and quality assurance procedures were applied according to the published guidelines (Broadhurst et al., 2018; Dudzik et al., 2018). The principal component analysis (PCA-X) examined the acquired data for signal drift, variation in QC samples, and possible outliers. Tight QC clustering was observed (Figure 2), indicating the reproducibility of the samples’ treatment and high precision of the analytical procedure. Two outlying observations on the PCA-X model evaluated by Hotelling’s T2 range plot were removed from further calculations. Variations within measurements were calculated for QCs and expressed as relative standard deviation (% RSD) with the cut-off value RSD >30%. Data were normalized according to tricosane (CAS Registry Number: 638-75-5, IUPAC Standard InChIKey: FIGVVZUWCLSUEI-UHFFFAOYSA-N). The calculations were performed using MATLAB scripts (Matlab R2015, Mathworks) and Excel (Microsoft). Multivariate analysis was performed using SIMCA-P + 16.0 software (Umetrics, Umea, Sweden). A combination of VIP-p (corr.) (correlation coefficient combined with VIP, variable influence on the projection) based on the orthogonal partial least squares discriminant analysis (OPLS-DA) model was applied for specified interpretations with the threshold for the variable selection set to VIP >1.0 and p(corr.) > .4. The Kolmogorov–Smirnov–Lillefors test verified data normality and the variance ratio, by Levene’s test. The Student *t-test statistical* significance level was 95% (*p* < .05), and the false discovery rate was .05. Univariate statistical analyses were performed with MATLAB scripts (Matlab R2015, Mathworks).

### Fatty acid analysis

The plasma cholesteryl esters were isolated as described previously (Astarita et al., 2014; Laiakis et al., 2014). Fatty acids were identified as the corresponding fatty acid methyl esters (FAMEs) and analyzed by gas chromatography as described earlier (Bukowiecka-Matusiak et al., 2018).

### Statistical analysis

The Shapiro–Wilk test was used to test for data normality. Continuous variables were presented as medians with corresponding interquartile ranges (IQRs). We used the Mann–Whitney rank test to compare the GDM patients and the healthy control group. The Benjamini–Hochberg procedure was applied to calculate the false discovery rate (FDR). In all analyses, *p*-values equal to or less than 0.05 were considered statistically significant. Furthermore, patients’ obtained biochemical and anthropometric characteristics were subjected to Spearman’s rank correlation analysis.

All statistical analyses were carried out using Statistica 13.1 (TIBCO Software, Palo Alto, CA) and the additional analytical extensions (StatSoft, Polska Sp. z.o.o., Poland).

## Results

The clinical and biochemical characteristics of the groups are given in Table 1. These results indicate that the studied groups did not differ significantly in age, insulin concentration, HOMA-IR, QUICKI, and LDL levels.

**TABLE 1:** Biochemical and clinical parameters of the GDM and NGT groups.

	GDM (*n* = 32)^a^	NGT (*n* = 11)^a^	*p*
Age	31.0 (28.5–34.0)	29.0 (28.0–30.0)	0.2003
BMI [kg/m^2^]	23.7 (21.6–27.5)	20.64 (19.6–21.6)	**0.0044**
FPG—OGTT 0’ [mg/dL]	85.0 (80.0–97.0)	75.80 (70.8–84.0)	**0.0067**
OGTT 60’ [mg/dl]	178 (150.0–190)	115.25 (113.3–162.3)	**0.0075**
OGTT 120’ [mg/dL]	160.0 (153.0–173.0)	99.6 (88.8–100.9)	**<0.0001**
CRP [mg/L]	3.4 (1.7–6.28))	1.5 (1.1–2.5)	**0.0366**
Insulin [µU/mL]	10.8 (7.8–17.4)	13.9 (7.0–15.5)	0.7710
HOMA-IR	2.2 (1.6–3.6)	2.3 (1.3–3.2)	0.5306
HOMA-ß	203.3 (147.3–241.9)(147.3–241.9)	287.3 (208.5–344.0)	*0.0506*
QUICKI	.34 (0.32–0.35)	.34 (0.32–0.37)	0.5306
Total cholesterol [mg/dL]	256.1 (217.8–283.3)	219.5 (191.5–242.0)	**0.0259**
HDL [mg/dL]	74.1 (58.7–87.3)	61.4 (53.9–67.1)	*0.0692*
LDL [mg/dL]	135 (108.0–169)	119.0 (105.0–139.0)	0.3272
TG [mg/dL]	205.8 (161.0–249.2)	157.6 (116.5–203.9)	*0.0618*

^a^ Median with IQR.

IQR, interquartile range; BMI, body mass index measured during the first visit; FPG, fasting plasma glucose; OGTT, oral glucose tolerance test; CRP, C-reactive protein; HOMA-IR, homeostasis model assessment of insulin resistance; HOMA-Β, homeostasis model assessment of beta cells; QUICKI, quantitative insulin sensitivity check index; HDL, high-density lipoprotein; LDL, low-density lipoprotein; TG, triglycerides.Bold values are statistically significant.

The following parameters achieved concentrations significantly higher in the GDM group in comparison to the control group: FPG (85 mg/dl vs. 75.8 mg/dl; *p* = .0067), OGTT 60’ (178 mg/dl vs. 115.25 mg/dl, *p* = .0075), OGTT 120’ (160 mg/dl vs. 99.6 mg/dl, *p* < .0001), CRP (3.4 mg/L vs. 1.5 mg/L, *p* = .0366), and total cholesterol (256.1 mg/dl vs. 219.5 mg/dl, *p* = .0259). The concentrations of triglyceride and HDL were higher in the GDM group. However, these differences were at a borderline of significance (205.8 mg/dl vs. 157.6, *p* = .0618; 74.1 mg/dl vs. 61.4 mg/dl, *p* = .0692; 287.3 vs. 203.3, *p* = .0506, respectively). HOMA presented the opposite trend-β, but this change was also at a borderline of significance (203.3 vs. 287.3, *p* = .0506). We observed notable differences between the medians for both HDL and HOMA-β, but they were not statistically significant, which may be explained by a difference in the number of subjects in the groups (GDM = 32 versus NGT = 11).

### Multivariate statistical analysis

**FIGURE 1:**
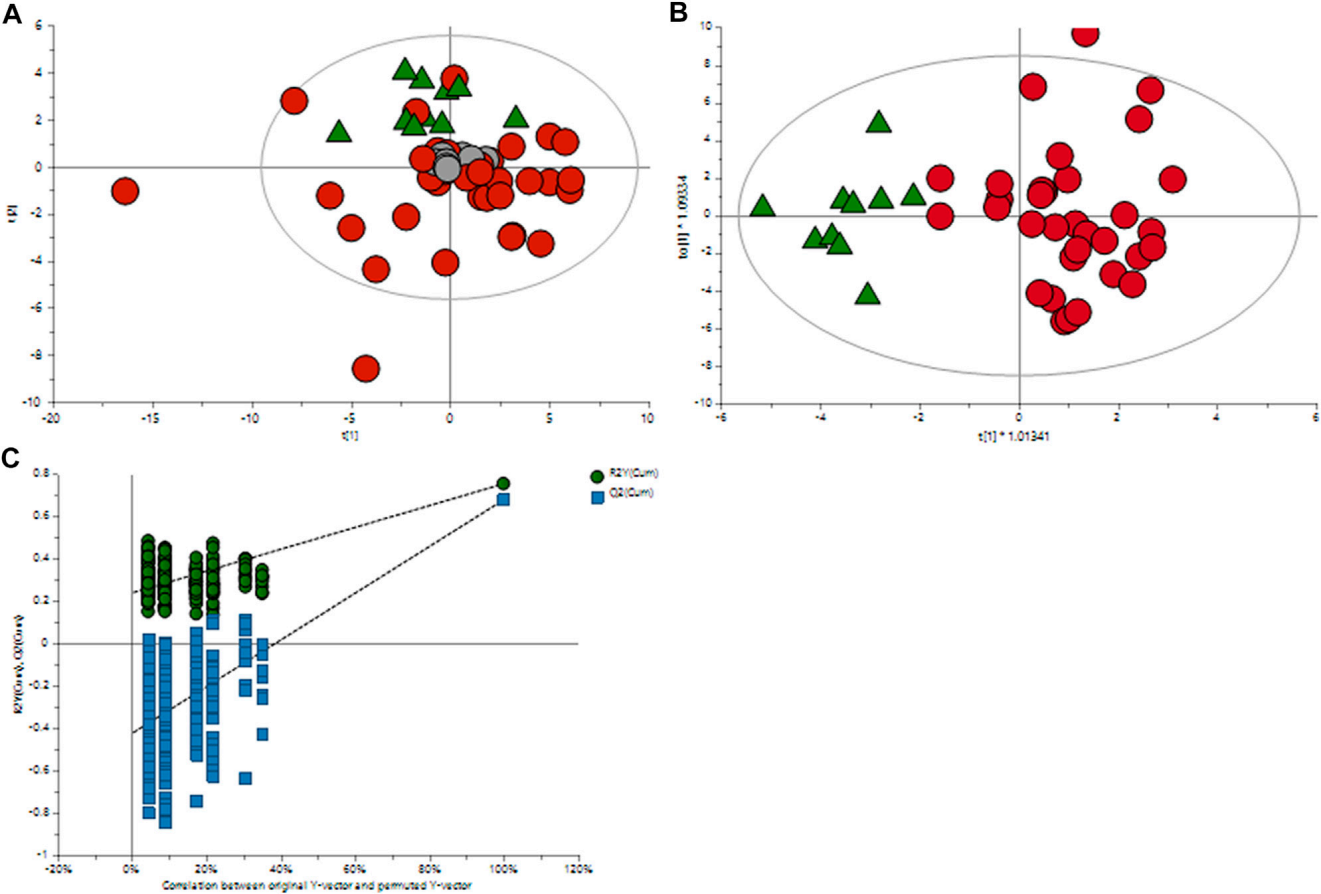
Multivariate OPLS-DA plots of plasma metabolomic profiles of GDM and NGT patients. **(A)** Principal component analysis (PCA) score plot of the metabolic profile of the NGT and GDM groups after mean-centering and not (Ctr) scaling. **(B)** OPLS-DA score plot of the lipid profile of the NGT and GDM groups after unit variance (UV) scaling. **(C)** Score plot of the OPLS-DA model obtained from **(B)**. The resulting R2 and Q2 values were plotted. The green dots represent the R2Y values obtained from the displacement test, the blue square dots represent the Q2 values obtained from the displacement test, and the two dashed lines represent the regression lines of R2Y and Q, respectively.

**FIGURE 2:**
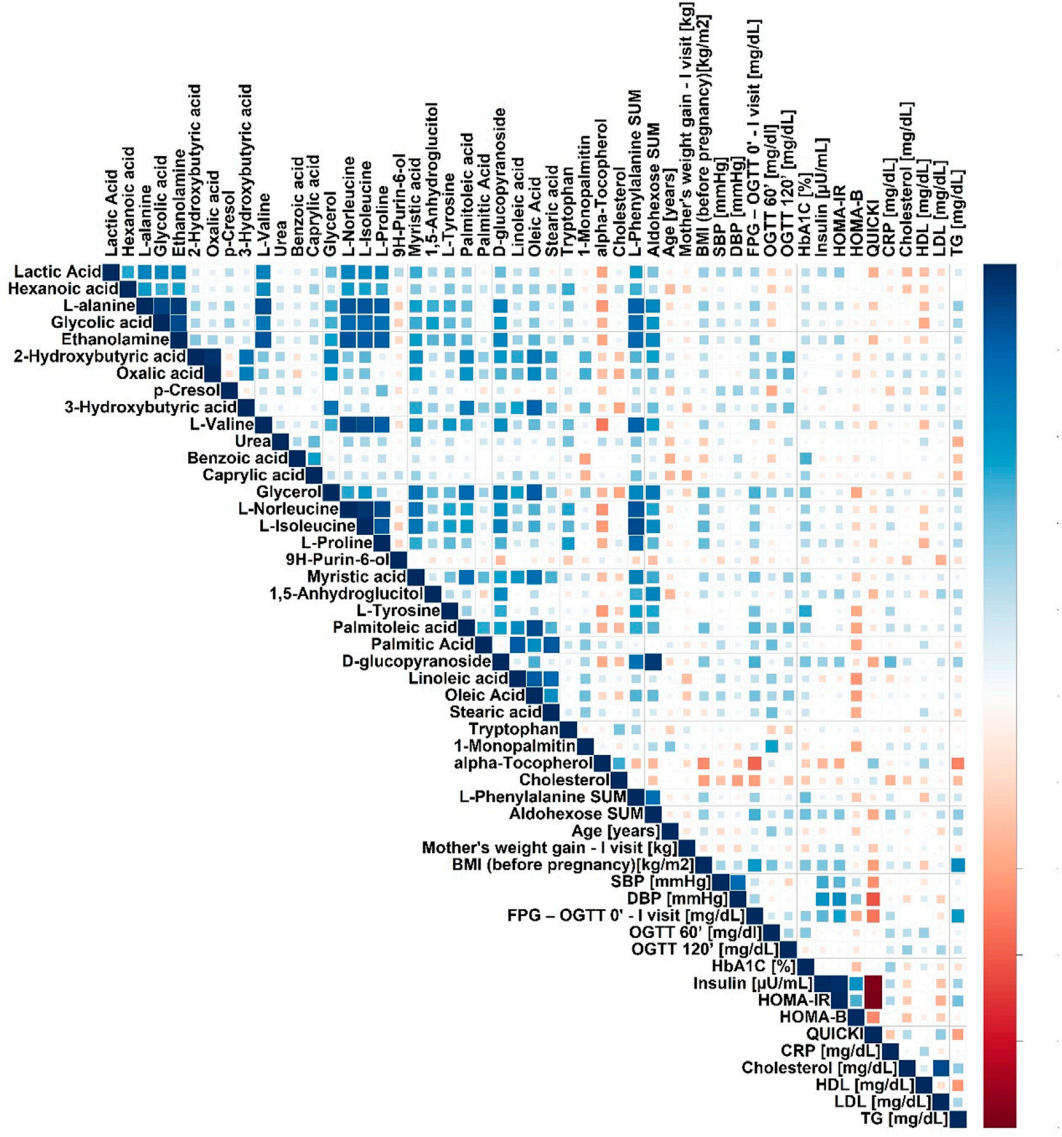
Spearman’s correlation heatmap for metabolites in plasma significantly changed in pregnant GDM versus NGT women.

The orthogonal partial least squares discriminant analysis was performed to screen for significantly differential metabolites, indicating the control and GDM groups’ separation (Figure 1) with an *R* ^*2*^ value of .76 and a predictive *Q* ^*2*^-value of .68. CV-ANOVA (*p*-value 5.12 E-09) demonstrated that the OPLS-DA model was highly significant. Additionally, internal cross-validation was conducted to test whether the OPLS-DA model was valid. A 200-step random pre-mutation was carried out, indicating no existed overfitting (Figure 2C). Six metabolites, namely, 2-hydroxybutyric acid, 3-hydroxybutyric acid, oxalic acid, glycerol, tryptophan, and aldohexose, were identified as significantly altered following the established multivariate VIP (>1.0) and p(corr) (> .05) criteria (Figure 1; Table 2). Subsequently, these metabolites were found to be statistically relevant in the independent *t*-test and false discovery rate (fdr)-adjusted *p*-value. The detailed results are shown in Table 2.

**TABLE 2:** Metabolites in the plasma significantly changed in gestational diabetic women compared to normoglycemic women.

Name	CV-QC	*p*(corr.)	VIP	JK	*p-value*	*P*BH	Δ(%)	FC
2-Hydroxybutyric acid	19	0.71	1.57	JK	0.00002	0.00015	135	2
Oxalic acid	19	0.73	1.59	JK	0.00006	0.00033	133	2
3-Hydroxybutyric acid	27	0.75	1.62	JK	0.00000	0.00001	505	6
Glycerol	15	0.64	1.50	JK	0.00000	0.00003	77	2
Tryptophan	9	−0.45	1.04	JK	0.03995	Ns	−31	1
Aldohexose	17.37	0.43	1.11	JK	0.00821	0.02711	40	1
	17.37							
	25.7							

Δ (%) change represents the increase (+) or decrease (−) of the mean in the GDM group versus NGT. In order to obtain a normal distribution, data were transformed by applying a log(base 2). CV-QC is defined as coefficient of variation of quality control. Statistical significance was reported as the value of multivariate analysis from the threshold for variable selection set to VIP>1.0, and predictive loading values p(corr) > .4 was applied. The Jack–Knife multivariate statistical analysis (JK) had confidence intervals estimated at 95% confidence level. Data were statistically significant according to univariate analysis with the Benjamini–Hochberg correction, where *p* < .05 was considered significant. FC is the fold change which describes the magnitude of changes between GDM and NGT groups.

We performed a correlation analysis between significantly changed metabolites and biochemical and anthropometrical data. The results are presented in Figure 2. Out of all analyzed metabolites, a moderate correlation of 2-hydroxybutyrate, 3-hydroxybutyrate, and oxalic acid, with OGTT-120’, was observed.

The results concerning the profile of cholesteryl esters are collected in Table 3. Among the 14 measured fatty acids treated as a representative fatty acid cluster contained in cholesteryl esters, myristic, oleic, arachidonic, and α-linoleic acids were significantly changed.

**TABLE 3:** Plasma cholesteryl ester profiling using 14 fatty acids as a representative cluster.

Fatty acid^a^	GDM^b^	NGT^b^	Δ(%)^c^	*p*	FDR
Saturated fatty acids (SFAs)					
Myristic (C14:0)	0.31 (0.24–0.42)	0.49 (0.33–0.57)	−37%	0.0121	0.0449
Palmitic (C16:0)	5.90 (5.24–6.86)	6.15 (5.11–7.10)	−4%	0.8271	0.8960
Stearic (C18:0)	0.40 (0.29–0.50)	0.36 (0.31–0.49)	+11%	0.6726	0.7949
Monounsaturated fatty acids (MUFAs)					
Sapienic (C16:1)	0.45 (0.35–0.57)	0.47 (0.36–0.82)	−4%	0.4658	0.7570
Palmitoleic (9c C16:1)	2.74 (2.05–3.32)	3.31 (2.53–4.34)	−17%	*0.0596*	0.1291
Oleic (C18:1)	21.38 (20.37–22.25)	22.44 (21.89–23.79)	−5%	0.0056	0.0361
Vaccenic (11c C18:1)	0.92 (0.84–1.04)	0.87 (0.76–0.93)	+6%	*0.0596*	0.1291
Polyunsaturated fatty acids (PUFAs)					
Linoleic (C18:2)	55.09 (52.49–58.25)	56.07 (51.69–58.85)	−2%	0.6726	0.7949
α-linolenic (n-3 C18:3)	1.0 (0.80–1.30)	1.24 (1.08–1.54)	−19%	0.0138	0.0449
γ-linolenic (n-6 C18:3)	0.69 (0.53–0.82)	0.72 (0.55–0.81)	−4%	0.9535	0.9535
ARA (C20:4)	8.75 (7.73–9.74)	6.78 (6.03–8.24)	+29%	0.0001	0.0017
EPA (C20:5)	0.66 (0.75–1.22)	1.12 (0.85–1.22)	−41%	0.9454	0.9672
DPA (C22:5)	0.68 (0.48–1.03)	0.61 (0.40–0.92)	+11%	0.6515	0.7949
DHA (C22:6)	0.68 (0.36–0.79)	0.51 (0.40–0.72)	+33%	0.4223	0.7569
ARA: DHA	14.46 (10.70–18.28)	15.36 (8.83–16.78)	−6%	0.9036	
LA: ARA	6.23 (5.60–6.96)	7.72 (7.22–8.94)	−19%	0.00006	

^a^ Cholesteryl esters are reported as relative percentages of fatty acid methyl esters (% rel.) obtained by gas chromatographic analysis and the calibration procedure after cholesteryl ester isolation and work-up, as previously reported. (Burzynska-Pedziwiatr et al., 2020) See *Materials and Methods* for the experimental details. FDR, false discovery rate.

^b^ Median with IQR.

^c^ Differences between the GDM and NGT groups; ARA, arachidonic acid; EPA, eicosapentaenoic acid; DPA, docosapentaenoic acid; DHA, docosahexaenoic acid.

Among SFAs, only the concentration of myristic acid tends to decrease in GDM patients with a statistical significance (∆ = −37%. *p* = .0121). The relative content of MUFAs tends to decrease with oleic acid significantly lowered (Δ = −5%. *p* = .0056), whereas vaccenic acid (C18:1) is increased in the plasma of the GDM group in comparison with the control group with borderline significance (Δ = 6%. *p* = .0596). In the case of PUFAs, n-6 arachidonic acid increases (Δ = 29%. *p* = .0001) and n-3 α-linolenic acid decreases (Δ = −19%. *p* = .0138), which was statistically significant (Table 3).

**FIGURE 3:**
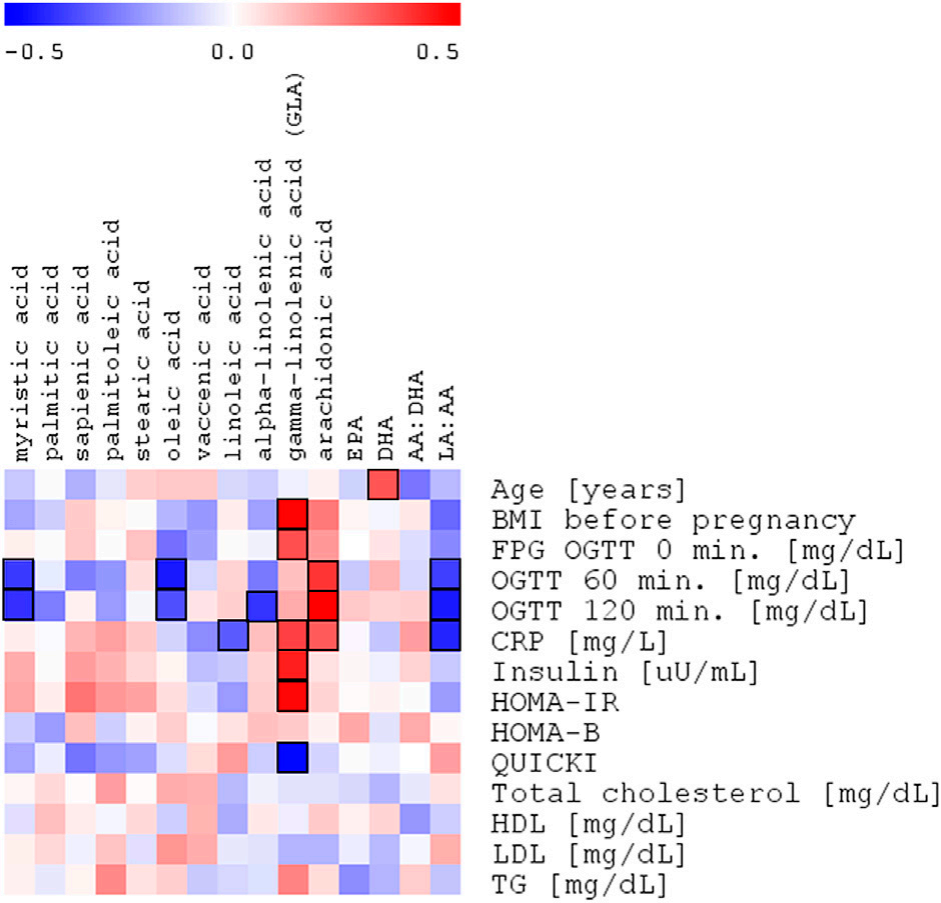
Spearman’s correlation heatmap of the changed fatty acids in cholesteryl esters with OGTT with OGTT 120’ glucose plasma concentration in the entire study group of pregnant women (NGT + GDM; *n* = 43).

In this analysis, both myristic acid and oleic acid contents were negatively correlated with OGTT 60’ (*R* = −.38. *p* = .0202 and *R* = −.42. *p* = .0058, respectively, Table 4; Figure 3). OGTT 120’ (*R* = −.39. *p* = .0088 and *R* = −.34. *p* = .0248) and the latter parameter having negative correlation with α-linolenic acid were reported (*R* = -0.39. *p* = .0090). Among all the measured cholesteryl esters, only arachidonic acid showed a positive correlation with OGTT 60’ (R = .39, *p* = .0157), OGTT 120’ (*R* = .50, *p* = .0005), and CRP (*R* = .32, *p* = .0198).

**TABLE 4:** Correlation with clinical data with top six changed cholesteryl esters—GDM and NGT together in the group.

	Myristic acid		Palmitoleic acid		Oleic acid		Vaccenic acid		α linolenic acid		ARA	
	*R*	*P*	*R*	*p*	*R*	*P*	*R*	*p*	*R*	*p*	*R*	*p*
Age [years]	−0.10	0.4547	−0.05	0.7306	0.11	0.4360	0.11	0.4509	−0.09	0.5009	0.03	0.8492
BMI^a^—before pregnancy	−0.17	0.2515	0.02	0.8910	−0.14	0.3597	−0.20	0.1894	−0.18	0.2251	*0.26*	*0.0873*
FPG—OGTT 0’ [mg/dL]	0.03	0.8417	−0.00	0.9971	−*0.28*	*0.0571*	−0.18	0.2257	−0.02	0.8936	0.20	0.1743
OGTT—60’ [mg/dL]	−**0.38**	**0.0202**	−0.21	0.2166	−**0.44**	**0.0058**	−0.08	0.6454	−0.26	0.1162	**0.39**	**0.0157**
OGTT—120’ [mg/dL]	−**0.39**	**0.0081**	−0.20	0.1985	−**0.34**	**0.0248**	0.05	0.7396	−**0.39**	**0.0090**	**0.50**	**0.0005**
CRP [mg/L]	0.04	0.7982	0.19	0.1800	−0.08	0.5544	−0.22	0.1074	0.13	0.3655	**0.32**	**0.0198**
Insulin [uU/mL]	0.16	0.2526	0.15	0.2836	0.02	0.8895	−0.13	0.3723	0.09	0.5397	0.06	0.6698
HOMA-IR	0.18	0.2438	0.20	0.1771	0.06	0.6803	−0.07	0.6506	0.10	0.5210	0.08	0.5929
HOMA-B	−0.09	0.5549	−0.09	0.5712	0.10	0.5312	−0.01	0.9636	0.12	0.4394	0.02	0.8823
QUICKI	−0.18	0.2438	−0.20	0.1771	−0.06	0.6803	0.07	0.6506	−0.10	0.5210	−0.08	0.5929
Total cholesterol [mg/dL]	0.02	0.8911	0.19	0.1800	0.16	0.2446	0.16	0.2683	−0.02	0.8652	−0.06	0.6966
HDL [mg/dL]	−0.06	0.6873	−0.02	0.9153	−0.09	0.5133	0.15	0.2921	0.04	0.7597	0.10	0.4822
LDL [mg/dL]	0.03	0.8299	0.12	0.4125	0.21	0.1419	0.16	0.2501	−0.06	0.6926	−0.14	0.3058
TG [mg/dL]	0.03	0.8471	0.23	0.1008	0.09	0.5113	−0.10	0.4725	−0.06	0.6756	0.07	0.6206

^a^ Assessed before pregnancy.

BMI, body mass index; HbA1C, glycated hemoglobin; HOMA-IR, homeostasis model assessment of insulin resistance; HOMA-β, homeostasis model of assessment of the β cell function.Bold values are statistically significant.

## Discussion

Here, we report the untargeted and targeted analyses of the plasma of pregnant women in the third trimester of pregnancy diagnosed with GDM against normoglycemic pregnant women as a control group (NGT).

In our study, among the identified plasma metabolites, the contents of 2-hydroxybutyric acid and 3-hydroxybutyric acid increased in GDM women compared with those in the NGT group (135%–505%, respectively; Table 2). We also observed higher levels of oxalic acid (133%), glycerol (77%), D-glucopyranoside (549%), and aldohexose (44%) and a lower level of tryptophan (−37%) in the GDM group.

2-hydroxybutyric acid could be derived from α-ketobutyrate, the product of amino acid catabolism (threonine and methionine) (Bui et al., 2019) or as the product of glutathione (GSH) anabolism obtained through the cysteine-formation pathway (Lord and Bralley, 2008). 2-hydroxybutyric acid is catabolized into propionyl-CoA, an essential product of the citric acid cycle responsible for energy supply in the form of ATP and carbon dioxide (Landaas, 1975). The higher concentration of 2-hydroxybutyric acid is associated with increasing oxidative stress, which can be connected with β-cell dysfunctions and an increase in free fatty acid (FFA) concentrations. All these factors are connected with hyperglycemia and insulin resistance (Varvel et al., 2014). Due to surplus oxidative stress, more GSH is produced through the cysteine anabolism, leading to the synthesis of α-ketobutyrate, which is converted to 2-hydroxybutyric acid.

On the other hand, FFAs, synthesized in excess under IR conditions, are oxidized in the tricarboxylic acid (TCA) cycle, leading to an excess of glutamate, alanine, and α-ketobutyrate production (Cobb et al., 2013; Ferrannini et al., 2013). In our study, the higher content of 2-hydroxybutyric acid was positively correlated with the OGTT test, as reported earlier (Dudzik et al., 2017). Our results corroborate previosly established role of 2-hydroxybutyric acid as a potential biomarker of glycaemic changes both, in GDM and T2DM (Xu et al., 2013; Padberg et al., 2014; Salek et al., 2020).

As mentioned previously, the elevated level of 2-hydroxybutyric acid among GDM women could be explained by the redox imbalance, leading to impairment in glutathione synthesis and increase in fatty acid oxidation. This status can result in a higher level of 3-hydroxybutyric acid, synthesized from fatty acids that circulate in the bloodstream during fasting, prolonged exercise, or carbohydrate restriction as one of the ketone bodies. 3-hydroxybutyric acid is also a partial degradation product of the branched-chain amino acids such as valine, isoleucine, and leucine, which are released from muscles for gluconeogenesis (Montelongo et al., 1992; Klein and Shearer, 2016). In our study, a higher concentration of 3-hydroxybutyric acid positively correlated with OGTT after 2 h.

Oxalic acid is engaged in the glycolytic pathway as a competitive inhibitor of lactate dehydrogenase (LH). This enzyme catalyzes the conversion of pyruvate to lactic acid. In our study, the level of oxalic acid is increased. Some studies in the literature suggest the association of elevated levels of these metabolites with kidney diseases (Mydlík and Derzsiová, 2008); however, such cases were not reported in our cohort.

One of the more interesting metabolites in our study is tryptophan, whose concentration in the GDM group was decreased. This amino acid is metabolized by the kynurenine pathway, which is the primary way for tryptophan degradation and leads to xanthurenic acid as one of the metabolites. This compound is one of the factors responsible for insulin resistance (Favennec et al., 2015). The study by Chen et al. (2016) reported that the concentration of tryptophan increased in obese people and can start to fall after nutrition intervention and when the weight returns to normal. Tryptophan is an essential amino acid, meaning the human body cannot synthesize it; therefore, tryptophan must be delivered with food. In pregnant women, this amino acid is also necessary for the developing fetus, which may explain the decreased level of this metabolite in our study group. Additionally, GDM women from the studied cohort were not obese, which may also cause differences between our results and those of the literature (Oxenkrug, 2013; Oxenkrug, 2015).

Glycerol and free fatty acids are the products of triacylglycerol lipolysis. The insulin-resistant state increases lipolysis and overproduction of these two metabolites (Hagen et al., 1963; Eckel et al., 2010). After lipolysis, glycerol is engaged as a gluconeogenic substrate in gluconeogenesis. In our cohort, we observed an increased level of this metabolite, which is typical in the presence of hyperinsulinemia. However, studies by Karpe et al. suggested that the levels of free fatty acids and glycerol are usually increased in the plasma of diabetes patients, despite only slightly affecting insulin resistance (Karpe et al., 2011).

In the reported study, among the 14 measured fatty acids, which were earlier considered representative of the most common types of fatty acids contained in this lipid class (Ferreri et al., 2016) (Bukowiecka-Matusiak et al., 2018), only the concentration of myristic acid tended to decrease in GDM patients with statistical significance (Table 2). There is growing evidence that fatty acids with different degrees of saturation affect insulin sensitivity and lipid/glucose metabolism in diverse ways (Palomer et al., 2018). The human LCAT has an affinity for fatty acids in the following order: 18:2n-6 > 18:1n-9 > 20:4n-6 > SFA, which can explain a higher proportion of 18:2 n-6 than 18:1n-9 in cholesteryl esters (CE) as a result of LCAT rather than acyl-CoA-cholesterol acyltransferase activity.

**FIGURE 4:**
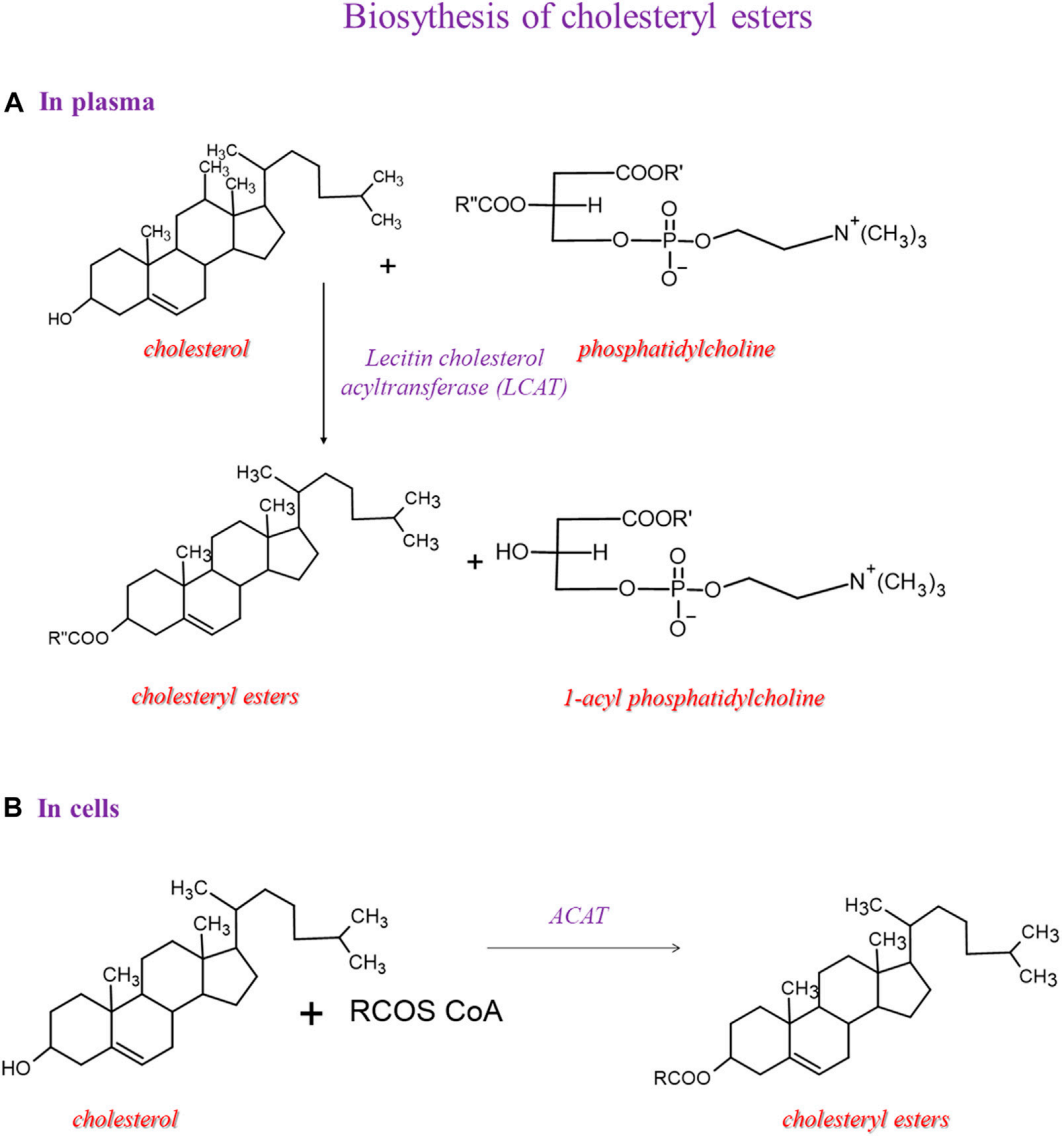
Biosynthesis of cholesteryl esters and then we showed 2 pathways: one in plasma and another in the cell.

Two enzymatic pathways lead to cholesteryl ester synthesis (Figure 4). Within the cells, the acyl-CoA-cholesterol acyltransferase (ACAT), specific for 18:1 n-9, catalyzes the conversion of cholesterol and acyl-CoA into cholesteryl esters. In the second pathway, which occurs in the plasma, the esters of cholesterol and fatty acids are obtained mainly due to the transfer of fatty acids from position 2 of the glyceryl moiety of phosphatidylcholine to cholesterol reaction is catalyzed by the LCAT.

The mechanism of action of LCAT takes place in two steps. LCAT binds to the lipoprotein, cleaves the fatty acid in the sn-2 position in phosphatidylcholine, and transfers it onto Ser181. In the next step, the fatty acid is trans-esterified to the 3-β-hydroxyl group on the A ring of cholesterol to form cholesteryl ester and lysophosphatidylcholine (Glomset, 1968). This reaction occurs for HDL; therefore, this process is essential in HDL maturation and reverses cholesterol transport to the liver. Because of the higher hydrophobicity of cholesteryl esters than cholesterol, the LCAT promotes the cellular removal of cholesterol (Tosheska Trajkovska and Topuzovska, 2017). In our cohort, the significant enrichment of arachidonic acid in cholesteryl esters gives information on a lipid class that has been considered with high attention for the relevance in lipoprotein formation during pregnancy, which is affected in GDM conditions (Herrera and Desoye, 2016). More work is needed to explain this increase in GDM, since it would be very interesting to deepen the significance of the metabolic shift of arachidonic acid between cholesteryl and membrane phospholipid structures, also in view of trimester-specific ranges of fatty acid contents in plasma lipids determined as the benchmark for the evaluation of the health of pregnant women (Martín-Grau et al., 2021).

Earlier, Takato et al. demonstrated that myristic acid (14:0) markedly increased diacylglycerol kinase δ (DGKδ) concentration in mouse C2C12 myotubes of the congenital type 2 diabetes model; For other fatty acids examined, such as lauric (12:0), palmitic (16:0), and stearic (18:0) acids, no influence was detected. Furthermore, myristic acid significantly augmented the glucose uptake in a DGKδ-related manner (Takato et al., 2017). DGKδ is an essential enzyme in the suppression of the pathogenesis of type 2 diabetes and its upregulation is important for the prevention and treatment of the disease. Iwata et al. revealed that myristic acid stabilizes DGKδ2 and mediates the attenuation of DGKδ2 protein degradation in skeletal muscle cells (Takato et al., 2017). The authors demonstrated that this process was fatty acid-specific, protein-specific, and cell type-specific. These findings shed light on the role of DGKδ2 in the pathogenesis of T2DM and provided novel insights into the molecular mechanisms of the disease (Iwata et al., 2019).

In our study, the concentration of myristic acid was decreased by about 37%, indicating that its deficiency may be related to insulin resistance and a reduced glucose uptake in GDM. This outcome is in line with the results of Ericson et al. (2015), who recently reported that a high intake of myristic acid (14:0) was significantly associated with a decreased risk of type 2 diabetes, while the consumption of palmitic acid (16:0) was not. It is worth noting that along with the insulin-dependent glucose uptake, myristic acid-enhanced insulin-independent glucose uptake was found in C2C12 mice myotubes (Wada et al., 2016). Therefore, myristic acid improved both insulin-dependent and -independent glucose uptakes in mice with type 2 diabetes. In our study, the negative correlation of myristic acid with OGTT 60’ and OGTT 120’ was observed, which may be connected with a diminished glucose uptake in GDM (Table 4; Figure 2).

In the plasma of the GDM group, in comparison with the control group, the relative content of MUFAs tends to decrease, except for vaccenic acid, which is increased. This change is not statistically significant (Table 2). Oleic acid (18:1cis-9) content was lower in the GDM group than in the NGT group. The observed negative correlations with OGTT 60’ and OGTT 120’ (*R* = −.44. *p* = .0058 and *R* = −.34 *p* = .0248, respectively) were observed, which may be connected with diminished glucose uptake in GDM (Table 4; Figure 3). Several mechanisms were proposed to explain the protective effects of oleic acid for T2DM (Coll et al., 2008) (Peng et al., 2011; Aa et al., 2017). However, it is still unclear whether an oleic acid-activated molecular mechanism is the most important for preventing insulin resistance, and there are still open questions regarding the effects of oleic acid on diabetes in humans (Palomer et al., 2018).

Furthermore, oleic acid may prevent the reduction of AMP-activated protein kinase (AMPK) caused by palmitic acid (Salvadó et al., 2013). Oleate prevents saturated fatty acid-induced ER stress, inflammation, and insulin resistance in skeletal muscle cells through an AMPK-dependent mechanism. The AMPK mechanism was used to develop a first-line drug in T2DM—metformin (Palomer et al., 2018). Kwon et al. suggested (Kwon and Querfurth, 2015) that oleic acid and metformin have comparable protective effects, both inhibiting the destructive effects of palmitic acid (Palomer et al., 2018). In our study, the decrease of oleic acid in the cholesteryl ester fraction may indicate an input in the pathophysiology of GDM, but this needs further investigation.

Previously, we determined the differences in the erythrocyte membrane lipid profiles of GDM women compared to the NGTs, revealing statistically significant changes in vaccenic and stearic acid contents (Bukowiecka-Matusiak et al., 2018). We postulated that the increased vaccenic acid concentration might indicate the increased metabolic transformation of palmitic acid (C16:0). This reaction is catalyzed with stearoyl-CoA-desaturase (Δ9-desaturase) and elongase in GDM women. Vaccenic acid can be transferred to the fetus, thus predisposing it to the enzymatic induction of the biosynthesis of palmitoleic acid (C16:1), which was earlier marked as a risk factor for metabolic abnormalities and new onset of diabetes (Serhan, 2008; Bukowiecka-Matusiak et al., 2018). Here, we report the study of plasma cholesteryl esters in the same population.

It is important to note that the fatty acid content as components of cholesteryl esters reflects, to a great extent, the consumption of dietary fats over the previous few weeks. Furthermore, this profile reflects the endogenous conversion of ingested fatty acids by desaturation and elongation or both (Katan et al., 1997). Diseases linked to insulin resistance are characterized by a specific fatty acid pattern in the serum lipid esters, storage lipids, and cell membranes of the skeletal muscles. In patients with disorders such as diabetes, obesity, and coronary heart disease, the fatty acid pattern is usually characterized by an increased content of palmitic acid (C16:0) and palmitoleic acid (C16:1), lower content of the essential linoleic acid (C18:2n-6 LA) and a higher ratio of dihomo-γ linolenic acid (DGLA, C20:3 n-6) in comparison to the matched healthy control subjects. There were no differences in ARA, EPA, and DHA between diabetic patients and controls (Vessby et al., 2002) (Boden and Shulman, 2002) (Mozaffarian et al., 2010).

In our study, the SFA concentrations were not elevated, and myristic acid was decreased in GDM versus NGT. Furthermore, in GDM among PUFAs, an increase in ARA and a decrease in ALA was observed, which stays in line with earlier findings (Ogundipe et al., 2020). According to the results of Ogundipe *et al*., GDM women had significantly elevated amounts of LA, which is parent to all n-6 PUFA, and decreased levels of DHA belonging to n-3 fatty acids. Moreover, GDM patients’ disorders in sequential n-6 metabolism, manifested in the decreased concentration of LA sequential fatty acid metabolites, were observed. Under physiological conditions, synthesis LA is converted to ARA (C20:4) in a sequence of elongase and desaturase-catalyzed reactions. Ogundipe et al. postulated that in GDM, an elevated ratio of LA/ARA might result from a reduced efficacy of the LA→ARA conversions due to insufficiencies in desaturase or elongase enzymes (Ogundipe et al., 2020).

Among our GDM group, the level of LA was practically unchanged, and DHA was not significantly increased, which may be related to the fact that our GDM patients were not as obese as patients in the study in Ogundipe *et al.* The LA/ARA ratio in GDM compared to NGT was statistically decreased (6.23 vs*.* 7.72 *p* = .0006; Table 2), but the ARA/DHA (14.46 vs. 15.36. *p* = .9036; Table 2) ratio was also reduced, but this change was not statistically significant.

During pregnancy, the activity of enzymes—∆6 and ∆5 desaturases responsible for n-3 and n-6 fatty acid conversions, is usually upregulated to increase the rate of hepatic ARA synthesis (Chalil et al., 2018). It is known that in diabetes, the activity of these enzymes is diminished due to insulin inefficiency or resistance. The explanation of the reduced ARA:DHA ratio in GDM may be the reason that the conversion of n-6 precursors into ARA in diabetic pregnant women may be uniquely impaired and affect the ARA status. This assumption may account for the prevalence of elevated n-6 precursors but reduce the ARA:DHA ratio in at-risk GDM women (Saether et al., 2003). In our study both, the LA:ARA and ARA:DHA ratios were lowered, which may be the result of the specificity of our GDM patients who were not obese. It is known that an elevated ratio of n-6/n-3 is associated with obesity, whereas a balanced ratio is essential in preventing obesity and weight gain (Simopoulos, 2016).

High BMI is one of the risk factors for GDM onset. The previous observational studies established that the diet of obese individuals is typically deficient in n-3 fats and rich in n-6 fats (Salek et al., 2020). Low maternal levels of parent n-3 fatty acids and those that are sequentially synthesized, including DHA, may play an essential role in the pathogenesis of GDM.

The imbalance between n-3 and n-6 fatty acids in the diet is mostly the problem of Western countries with a traditional diet based on vegetable oils such as corn, soybean, and sunflower, lacking in leafy green vegetables rich in n-3 fatty acids such as ALA and in fish oils which contain EPA and DHA belonging to long-chain PUFAs. The first group primarily contains n-6 PUFAs, including LA, which in humans is metabolized to DGLA, docosapentaenoic acid (DPA), and ARA.

PUFAs are essential fatty acids, meaning they cannot be synthesized *de novo* and must be taken from the food, and more importantly, the human body cannot interconvert them between n-3 and n-6 fatty acids. Therefore, once absorbed, the PUFAs are transported through the bloodstream to all tissues and organs in different forms (unesterified or esterified fatty acids), e.g., phospholipids, cholesteryl esters, or triacylglycerols, and are metabolized into bioactive species. However, it seems that the overall balance between n-3 and n-6 fatty acids is responsible for the modulation of several biological processes, including inflammation (Serhan, 2008; Dalli and Serhan, 2013). Arachidonic acid obtained from LA is incorporated into membrane phospholipids, released upon the action of phospholipase A1 and converted by cyclooxygenase (COX) into pro-inflammatory molecules, mainly prostaglandins and leukotrienes (Long et al., 2016; Miles and Calder, 2017). Pregnancy is a unique period for the woman’s body. This physiological process is where oxidative stress intensifies, which, in turn, is associated with an increased ROS production (Williams, 2003) and an increased level of ARA and its mediators (Cheng and Stewart, 2003). Additionally, pregnancy complicated with diabetes is associated with an increase in oxidative stress and the level of ARA (Williams, 2003).

Even if GDM patients involved in this study were not obese, the incorrect diet, rich in n-6 fatty acids, could rationalize the increased level of arachidonic acid (8.75 vs*.* 6.78 *p* = .0001) in our studies.

Although reliable from the point of view of statistical evaluation, the reported study has certain limitations resulting from a relatively small group of patients. In some cases, we observed substantial discrepancies between the medians. However, they were not statistically significant, which may be explained by large differences in the number of subjects in the groups. Consequently, we applied a strict FDR approach to the analysis in order to reduce the possibility of false-positive or -negative correlations.

**FIGURE 5:**
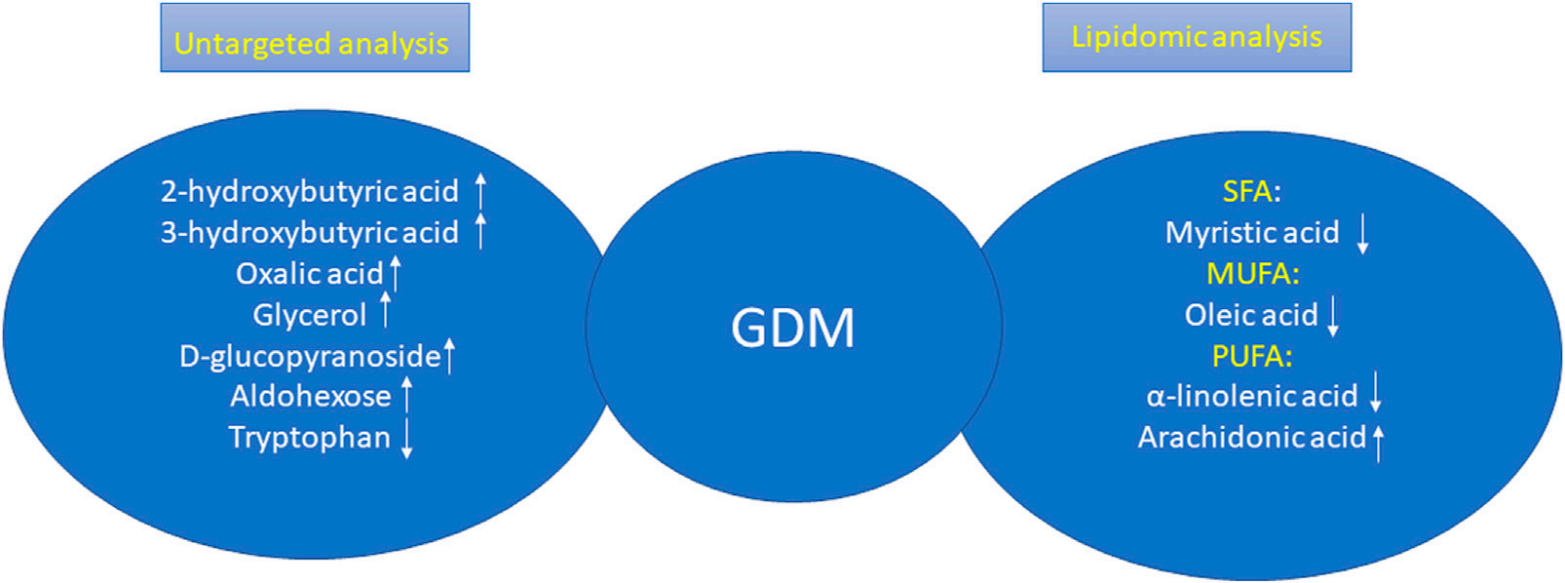
Changes in GDM women according the results obtained during untargeted analysis and lipidomic analysis.

To conclude, in our studies, we carried out a plasma untargeted analysis of GDM women vs. NGT ones and additionally the lipidomic analysis in order to profile the cholesteryl esters in both groups to attempt to search for early predictors of GDM. Among metabolites that resulted during untargeted analysis, only tryptophan was decreased in the GDM group, and the rest of the metabolites were increased including 2-hydroxybutyric acid, 3-hydroxybutyric acid, oxalic acid, glycerol, D-glucopyranoside, and aldohexose. In turn, among the lipid metabolites, we observed changes in all groups of fatty acids. All lipids, excluding arachidonic acid, belonging to PUFAs were decreased in the GDM group. These relationships are presented in Figure 5.

One can also assume the following limitation of the presented study. Fatty acid profiles were measured only at one point (24 ± 28 weeks), we had a small sample size during pregnancy, and the fatty acids were not followed up further. To address this, our further studies are designed to enroll a significantly higher number of participants to examine the lipid profiles of pregnant women at three time points of pregnancy and follow-up to explore the dynamics of changes in cholesteryl esters during and after pregnancy.

## Data Availability

The raw data supporting the conclusion of this article will be made available by the authors, without undue reservation.
